# Non-invasive assessment of liver fibrosis staging in chronic hepatitis B patients: combining two-dimensional shear wave elastography with serum indicators

**DOI:** 10.3389/fmed.2025.1709007

**Published:** 2026-01-12

**Authors:** Yaoren Zhang, Jianjun Yang, Qinyun Wan, Lu Gan, Jianxue Liu

**Affiliations:** 1 Department of Ultrasound Medicine, Baoji Central Hospital, Baoji, Shaanxi, China; 2 Department of Infectious Disease, Baoji Central Hospital, Baoji, Shaanxi, China

**Keywords:** elasticity imaging techniques, hepatitis B, liver cirrhosis, liver stiffness measurement, noninvasive diagnostics, serum markers

## Abstract

**Objective:**

This study aimed to evaluate the diagnostic accuracy of a novel approach that combines noninvasive indicators with two-dimensional shear wave elastography (2D SWE) to assess liver fibrosis stages in patients with chronic hepatitis B (CHB), using the Scheuer score as the reference standard.

**Methods and materials:**

2D SWE and serum indicators are commonly used for the noninvasive assessment of liver fibrosis. A total of 102 patients with CHB underwent 2D SWE measurements and serum tests for liver fibrosis markers. Standardized protocols were followed for all diagnostic procedures to ensure reproducibility and consistency. Binary logistic regression was used to generate a combined predictive probability value. Receiver operating characteristic (ROC) curves were used to evaluate the efficacy of the noninvasive diagnosis of liver fibrosis stages.

**Results:**

Combining multiple serum indicators increased the AUC for diagnosing substantial liver fibrosis from 0.735 (0.650–0.810) to 0.825 (0.746–0.888) (*p* = 0.016), for severe liver fibrosis from 0.815 (0.730–0.881) to 0.881 (0.810–0.932) (*p* = 0.024), and for cirrhosis from 0.904 (0.823–0.923) to 0.954 (0.900–0.984) (*p* = 0.013). The AUC for diagnosing substantial liver fibrosis increased from 0.805 (0.725–0.858) to 0.889 (0.820–0.939) (*p* = 0.040) with the combined serum indicators and 2D SWE, indicating a clinically meaningful improvement in diagnostic accuracy.

**Conclusion:**

The combination of multiple noninvasive indicators can improve the accuracy of assessing liver fibrosis stages in patients with CHB. This approach offers a potential noninvasive alternative to liver biopsy for assessing liver fibrosis, particularly in advanced stages that require clinical intervention.

## Introduction

Hepatitis B virus (HBV) infection is a worldwide epidemic, affecting approximately 257 million patients with chronic infections. In 2015, approximately 887,000 people died from HBV-related diseases, with cirrhosis and primary liver cancer accounting for 52.0 and 38.0% of those deaths, respectively (1). It is estimated that the prevalence of hepatitis B surface antigen (HBsAg) in the general population in China is currently between 5.0 and 6.0%, with approximately 70 million HBV infections in China and substantial mortality associated with liver fibrosis complications. Accurate, noninvasive diagnostic tools are critical for timely intervention and management (2, 3).

Accurate assessment of liver fibrosis stage in patients with chronic hepatitis B (CHB) is essential for disease evaluation, treatment guidance, and outcome monitoring. The presence of substantial fibrosis (≥S2) serves as a critical indicator for initiating antiviral therapy, particularly in CHB patients with persistent serum HBV-DNA positivity. Early detection of liver fibrosis in individuals with normal aminotransferase levels can effectively prevent disease progression and facilitate timely antiviral intervention, thereby addressing a major clinical challenge in CHB management (4).

The histopathological examination of liver biopsy (LB) is the gold standard for assessing liver fibrosis; however, LB is an invasive procedure that may cause pain in approximately 25% of patients. Furthermore, approximately 0.3–0.6% may experience severe complications, including bleeding, infection, or even death (5, 6). Therefore, some patients decline this examination, especially the second LB, which limits its clinical use. The LB tissue represents only 1/50,000 of the entire liver. A small sample may lead to underestimating the fibrosis stage, with missed liver cirrhosis ranging from 15 to 30%. Inadequate specimen length and fragmentation can lead to false positives and false negatives in diagnosing liver fibrosis (7–9).

Furthermore, variations among pathologists can affect diagnostic accuracy (10). Consequently, the noninvasive assessment of liver fibrosis has emerged as a significant area of interest and a challenging issue in contemporary research. Noninvasive methods for assessing liver fibrosis include imaging tests, elastography, and serum markers. Although imaging tests such as B-mode ultrasound, plain computed tomography (CT), and magnetic resonance imaging (MRI) can diagnose typical cirrhosis, they have low accuracy for evaluating liver fibrosis (11).

Serum indicators include blood markers and their corresponding algorithms (12–18). Serological tests require only a small amount of blood from patients. They offer several advantages, including ease of use, low cost, minimal invasiveness, and improved patient compliance. The majority of serum markers were originally developed using models established in patients with hepatitis C. These indicators are valuable only for patients with severe fibrosis, and their ability to predict mild to moderate liver fibrosis or fibrosis from other causes is unsatisfactory (19). Elastography, particularly shear-wave elastography using ultrasound techniques such as 2D SWE, acoustic radiation force (ARFI), Sound Touch Elastography (STE), and VCTE (Fibroscan), directly measures shear-wave velocity, which is related to tissue stiffness (20). Unlike VCTE, ARFI, 2D SWE, and STE, which can create real-time, two-dimensional images of liver stiffness, conventional ultrasound systems can provide both anatomical and stiffness information. While these techniques are highly valuable for diagnosing liver cirrhosis and may even substitute for LB, no noninvasive indicator can replace the pathological diagnosis provided by LB when assessing substantial liver fibrosis (21–24). No noninvasive method can replace the pathological diagnosis provided by LB when assessing significant liver fibrosis.

Combining elastography with serum markers leverages the strengths of both techniques, addressing limitations such as the low specificity of serum markers and variability in imaging results. To date, no studies have systematically evaluated the diagnostic accuracy of combining 2D SWE with multiple serum markers specifically for CHB patients, a population distinct from hepatitis C cohorts. This study aims to improve the diagnostic accuracy of liver fibrosis assessment by integrating 2D SWE with serum markers, providing a noninvasive, cost-effective diagnostic strategy that reduces reliance on invasive biopsies.

## Materials and methods

### Study populations

This prospective study was approved by the ethics committee of Baoji Central Hospital, and informed consent was obtained from all patients. All methods were performed in accordance with the Declaration of Helsinki. The inclusion criteria required patients to have hepatitis B DNA virus and HBsAg-positive serum for more than 6 months, to have not received antiviral treatment previously, and to have consented to an LB. The exclusion criteria included CHB patients with drug-induced hepatitis, hepatitis C, fatty liver, and those who experienced failure with SWE or VCTE. From June 2021 to April 2023, we selected 121 eligible patients from the Department of Infection at Baoji Central Hospital. Among them, 2 cases had CHB combined with CHC, and 5 cases had fatty liver disease. A total of 12 cases had failed measurements of 2D SWE and/or VCTE due to patient-related issues, including obesity, difficulty with breath-holding, and skin lesions, resulting in the exclusion of 19 patients from the study. The mean age of the 102 patients was 35.8 ± 12.3 years, ranging from 14 to 70 years, and the body mass index was 20.8 ± 2.0. There were 48 men with a mean age of 32.8 ± 10.9 years, ranging from 14 to 56 years, and 54 women with a mean age of 38.6 ± 13.0 years, ranging from 19 to 70 years. In the overall cohort of 102 patients, 60% of patients from each liver fibrosis stage were randomly assigned to the training cohort, totaling 61 cases. In contrast, the remaining 41 cases were allocated to the validation cohort.

### Liver stiffness measurements using 2D SWE

Liver stiffness was measured by 2D SWE on the day of LB using the new Aixplorer® imaging system (Supersonic Imagine, Aix-en-Provence, France) and a broadband convex array probe (SC6-1) with a frequency of 1–6 MHz. Point shear wave elastography (pSWE) and magnetic resonance elastography (MRE) are alternative options, but pSWE has a smaller sampling area than 2D SWE, and MRE is more expensive and time-consuming (25).

The Aixplorer® ultrasound system was selected for its advanced imaging capabilities, including high-resolution imaging and real-time elastography, both of which are essential to our study’s objectives. The SC6-1 probe is specifically designed for abdominal imaging, providing optimal frequency and penetration depth for the tissues we examined.

2D SWE examination was completed prior to LB. Patients were required to fast for more than 2 to 3 h before the measurement. Patients lay in a supine position and held their head with their right hand. The probe was then positioned in the intercostal space for scanning, resulting in clear B-mode images of the S5 and S6 segments of the liver. To initiate 2D SWE, select the mode. Then set the 2D SWE option to ‘Pen,’ adjust the opacity to 50%, and set the elasticity range to 30 kPa. The 2D SWE sampling frame was placed more than 1.0 cm beneath the liver capsule, measuring 4.0 × 3.0 cm, and was positioned to avoid intrahepatic blood vessels and focal liver lesions. Patients were instructed to hold their breath for 3–5 s at rest. After stabilizing the elastic graph, we proceeded to record liver stiffness values.

The region of interest (ROI) was marked using a Q-box™ (diameter: 2.0–3.0 cm) and placed at the position where the elastic graph signal was uniform. The same part of each patient was measured five times, and the median was calculated. We considered measurements with Emin < 0.2 kPa as failures because this threshold indicates insufficient pressure to provide reliable data for our analysis. Similarly, an IQR/M ratio greater than 30% indicates high variability in the measurements, which can compromise data consistency and reliability. In our analysis, we found that these criteria were met in approximately 10% of the excluded cases (26).

### Liver stiffness measurements using VCTE

VCTE was required to be completed within 1 week prior to LB to minimize potential variability in liver stiffness measurements. VCTE procedures were performed by the same well-trained technician to minimize inter-operator variability. The patient was supine, with their right hand holding their head to expand the intercostal space. The probe was positioned in the intercostal space between the 7th and 9th ribs, between the right midaxillary line and the anterior axillary line. Ten effective echoes were sequentially obtained from the same area. The median (M) of these echoes was recorded in kPa. Detection was deemed successful if the ratio of effective detection time to total detection time was greater than or equal to 60%, and the interquartile range to median ratio (IQR/M) was less than or equal to 30% (27).

### Serum markers of liver fibrosis

Serum marker measurements were performed under standardized conditions in accordance with the guidelines of the International Federation of Clinical Chemistry. The detection of all serum markers required fasting for more than 8 h and was performed within 1 week of the 2D SWE measurement. Serum markers included albumin (ALB), alkaline phosphatase (ALP), alanine aminotransferase (ALT), aspartate aminotransferase (AST), gammaglutamyl transferase (GGT), total bilirubin (TB), direct bilirubin (DB), indirect bilirubin (IB), total cholesterol (TC), hyaluronic acid (HA), type IV collagen (CIV), laminin (LN), platelets (PLT), international normalized ratio (INR), prothrombin (PT), α2-macroglobulin, gammaglobulin, apolipoprotein A1 (apo-A1), hepatitis B e antigen (HBeAg), and hepatitis B virus DNA. Aminotransferase levels have an upper normal limit of 40 U/L, while the normal platelet count range is 100 to 300 × 10^9^/L.

The seven serum models were calculated as follows: ① APRI score = [(AST/ULN) × 100]/platelet count 10^9^/l; ② Forns score = 7.811–3.131 × ln [platelet count (10^9^/l)] + 0.781 × ln [GGT (IU/l)] + 3.467 × ln [age(years)] – 0.014 [cholesterol (mg/dl)]; ③ FIB-4 score = [age (years) × AST (IU/l)]/[platelet count (10^9^/l) × ALT(IU/l)^1/2^]; ④ King score = age (years) × AST (IU/l) × INR/platelet count (10^9^/l); ⑤ FibroIndex = 1.738–0.064 × platelet count (10^9^/l) + 0.005 × AST (IU/l) + 0.463 × gammaglobulin; ⑥ Hepascore = y/(1 + y), y = exp. [− 4.185818 – (0.0249 × age) + 0.7464 × sex] + (1.0039 × a2-macroglobulin) + (0.0302 × HA) + (0.0691 × bilirubin) – (0.0012 × GGT); ⑦ RPR = RDW (%)/platelet count (10^9^/l).

These markers were selected based on the existing literature and our previous research. Furthermore, the serum indicators incorporated into these models are straightforward and readily accessible, enhancing their practical utility. Although some of these indicators were originally developed for chronic hepatitis C, they have since been validated in studies involving patients with hepatitis B (28–31).

### Liver biopsy and pathological analysis

Ultrasound-guided LB was performed on the S5 or S6 liver segments. Biopsy specimens less than 15 mm in length or with fewer than six portal tracts (except in cases of cirrhosis) were excluded. The specimens were fixed in formalin, embedded in paraffin, and stained with Masson’s trichrome, reticular fiber, and H&E.

Liver fibrosis assessment was performed by two pathologists with more than 10 years of work experience. We implemented a double-check system in which any discrepancies in their findings were reviewed and resolved by consensus. According to the Scheuer scoring system, liver fibrosis was divided into S0-S4 stages: stage 0 [S0], no hepatic fibrosis; stage 1 [S1], expansion of portal fibrosis; stage 2 [S2], fibrosis around the portal or a few fibrous septa; stage 3 [S3], a large number of fibrous septa with lobular structural disorder, no cirrhosis; stage 4 [S4], cirrhosis. Liver fibrosis stages ≥ S2 were defined as substantial fibrosis, and stages ≥ S3 were defined as severe fibrosis (32).

### Statistical analysis

All data were analyzed using SPSS 13.0 (SPSS Inc., Chicago, IL, USA) and MedCalc version 15.2.1 (MedCalc Program, Ostend, Belgium). A t-test or Mann–Whitney test was used for quantitative data comparison, and a *χ*^2^ test or Fisher’s exact test was used for qualitative data comparison. The test method was selected according to the data characteristics. Spearman’s test was used to evaluate the correlation between noninvasive indicators and the pathological stages of liver fibrosis. All noninvasive tests were included in binary logistic regression analyses using the forward stepwise method, with variables entered if *p* < 0.05. The 2D SWE values and single or multiple serum markers were analyzed using binary logistic regression analyses to calculate predictive probability values as combined predictors. The diagnostic performance of the 2D SWE, serum markers, and combined predictors was assessed using receiver operating characteristic (ROC) curves. The thresholds for sensitivity and specificity were selected using the Youden index, which maximizes the sum of sensitivity and specificity in predicting individual stages. A *p*-value of < 0.05 was considered statistically significant.

## Results

Among the 102 patients, there were 3 in S0, 33 in S1, 28 in S2, 18 in S3, and 20 in S4. Age, PCIII, CIV, LN, HA, PLT count, TB, DB, ALT, AST, GGT, ALP, ALB, and *γ*-globulin levels differ among the four stages of liver fibrosis. Age, CIV, LN, HA, TB, DB, ALT, AST, GGT, ALP, and γ-globulin levels are significantly higher in stage S4 than in stages S0–1, S2, and S3. In stage S4, PLT count and ALB levels are significantly lower than in stages S0–1, S2, and S3. The level of PCIII is significantly higher in stage S2 than in stages S0–1, S3, and S4. Gender ratio, BMI, red blood cell distribution width, INR, serum HBV DNA, IB, TC, and α2-macroglobulin levels were not significantly different among the different stages of liver fibrosis (Table 1).

**Table 1:** Clinical and laboratory findings.

Variable	S0-1 (*n* = 36)	S2 (*n* = 28)	S3 (*n* = 18)	S4 (*n* = 20)	*p-*value
Age	31.5 ± 12.4	33.3 ± 10.2	36.1 ± 12.7	48.1 ± 12.7	<0.001**
Male (female)	16 (20)	15 (13)	10 (8)	9 (11)	0.808
BMI (kg/m^2^)	20.7 ± 2.2	20.5 ± 2.5	20.8 ± 2.1	21.0 ± 1.2	0.563
HBV DNA (log_10_IU/mL)	7.51 (4.41–8.21)	7.29 (3.70–8.22)	5.14 (4.35–6.73)	6.71 (6.10–7.47)	0.610
HBeAg (+/−)	24 (11)	16 (12)	9 (9)	15 (5)	0.395
Procollagen type III (ng/mL)	42.6 (33.5–95.6)	82.1 (38.2–102.5)	38.9 (1.5–112.6)	33.3 (4.0–310.4)	0.041*
Collagen type IV (ng/mL)	46.5 (33.8–74.3)	67.2 (39.8–107.6)	67.9 (28.0–140.7)	131.4 (89.5–193.1)	0.010*
Laminin (ng/mL)	76.8 (44.3–106.8)	62.2 (30.2–130.1)	40.5 (22.6–91.3)	111.4 (54.36–246.7)	0.039*
Hyaluronic acid (ng/mL)	75.0 (56.2–85.5)	55.2 (44.1–72.6)	33.0 (14.6–99.1)	93.7 (69.1–177.5)	<0.001**
Platelet count (10^9^/L)	177.5 ± 59.8	161.0 ± 53.0	142.9 ± 44.1	92.7 ± 46.1	<0.001**
RDW (%)	13.23 ± 1.93	13.31 ± 1.34	13.6 ± 2.28	14.08 ± 2.44	0.646
Total bilirubin (μmol/L)	8.87 ± 4.36	10.95 ± 3.47	10.49 ± 3.60	15.56 ± 5.53	<0.001**
Direct bilirubin (μmol/L)	3.75 ± 1.55	4.48 ± 1.26	4.73 ± 1.40	8.22 ± 3.22	<0.001**
Indirect bilirubin (μmol/L)	5.09 ± 3.00	6.46 ± 2.43	5.76 ± 2.32	7.34 ± 2.66	0.070
ALT (IU/L)	26.89 ± 19.56	32.22 ± 19.87	34.61 ± 17.22	49.56 ± 33.36	0.010*
AST (IU/L)	22.91 ± 80.1	25.22 ± 9.66	27.94 ± 5.60	65.72 ± 53.30	<0.001**
GGT (IU/L)	17.37 ± 10.52	19.81 ± 13.17	21.06 ± 7.39	94.33	<0.001**
Alkaline phosphatase (IU/L)	84.89 ± 45.70	72.15 ± 28.10	62.22 ± 22.30	115.22 ± 90.79	0.010*
Albumin (g/L)	40.98 ± 2.70	40.48 ± 8.06	40.12 ± 3.08	37.31 ± 4.06	0.039*
Cholesterol (mg/dL)	3.84 ± 0.97	3.70 ± 0.94	3.56 ± 0.341	3.56 ± 0.72	0.573
Prothrombin time (%)	99.99 ± 11.33	97.20 ± 11.89	98.22 ± 9.48	92.69 ± 16.89	0.242
INR	1.02 ± 0.08	1.04 ± 0.08	1.02 ± 0.08	1.08 ± 0.12	0.321
a2-macroglobulin	8.62 ± 1.69	7.59 ± 1.40	8.06 ± 2.03	8.23 ± 1.09	0.164
*γ*-globulin	17.62 ± 3.20	17.44 ± 3.03	18.46 ± 3.31	23.41 ± 4.01	<0.001**
apolipoprotein-A1	1.23 ± 0.25	1.24 ± 0.27	1.35 ± 0.23	1.17 ± 0.31	0.559
VCTE	5.43 ± 3.59	6.02 ± 2.72	11.26 ± 3.86	21.8 ± 11.7	<0.001**
2D SWE	8.43 ± 6.23	8.85 ± 3.08	13.92 ± 3.83	23.82 ± 12.0	<0.001**

BMI, body mass index; HBV, hepatitis B virus; HBeAg, hepatitis B e antigen; ALT, alanine aminotransferase; AST, aspartate aminotransferase; GGT, gamma-glutamyl transpeptidase; INR, international normalized ratio; PCIII, procollagen type III; CIV, collagen type IV; LN, laminin; HA, hyaluronic acid.

Data are the mean ± standard deviation or median (interquartile range) or numbers of patients, when appropriate.

**Correlation is significant at the 0.01 level (two-tailed).

*Correlation is significant at the 0.05 level (two-tailed).

### Correlation between noninvasive indicators and liver fibrosis stage

The liver stiffness measured by 2D SWE had the strongest correlation with liver fibrosis stage (*r* = 0.779, *p* < 0.001), followed by VCTE (*r* = 0.719, *p* < 0.001), Forns score (*r* = 0.566, *p* < 0.001), APRI (*r* = 0.551, *p* < 0.001), King’s score (*r* = 0.548, *p* < 0.001), FibroIndex (*r* = 0.528, *p* < 0.001), FIB-4 (*r* = 0.492, *p* < 0.001), RPR (*r* = 0.425, *p* < 0.001), CIV (*r* = 0.348, *p* < 0.001), LN (*r* = 0.200, *p* = 0.035), PCIII (*r* = 0.162, *p* = 0.041), and Hepascore (*r* = 0.148, *p* = 0.048), successively. The HA had no obvious correlation with the liver fibrosis stage (*r* = 0.095, *p* = 0.298) (Table 2).

**Table 2:** Spearman’s coefficients for the noninvasive methods and liver fibrosis stages in patients with CHB.

Noninvasive parameter	Liver fibrosis stage	Spearman coefficient	*p*-value
2D SWE	Scheuer scores	0.779	< 0.001**
VCTE		0.719	< 0.001**
Forns score		0.566	< 0.001**
APRI		0.551	< 0.001**
King’s score		0.548	< 0.001**
FibroIndex		0.528	< 0.001**
FIB-4		0.492	< 0.001**
RPR		0.425	< 0.001**
CIV		0.348	< 0.001**
LN		0.200	0.035*
PCIII		0.162	0.041*
Hepascore		0.148	0.048*
HA		0.095	0.298

2D SWE, Two-dimensional shear wave elastography; APRI, Aspartate transaminase-to-platelet ratio index; FIB-4, Fibrosis index based on the four factors; RPR, Red cell distribution width-to-platelet ratio; CIV, Type IV collagen; HA, Hyaluronic acid.

**Correlation is significant at the 0.01 level (two-tailed).

*Correlation is significant at the 0.05 level (two-tailed).

### Comparison of 2D SWE and VCTE in the diagnosis of liver fibrosis stage

Among the 102 CHB patients, 84 cases were also successfully treated with VCTE. The pathological results for these 84 cases were as follows: 3 cases in S0, 34 cases in S1, 22 cases in S2, 12 cases in S3, and 13 cases in S4.

The AUCs for diagnosing substantial liver fibrosis, severe liver fibrosis, and cirrhosis with 2D SWE and VCTE were 0.805 vs. 0.802, 0.945 vs. 0.945, and 0.970 vs. 0.956, respectively. There was no significant difference in the AUC for liver fibrosis stage diagnosis between the two methods.

### Evaluation of liver fibrosis staging using 2D SWE combined with single or multiple serum indicators

The results of the binary logistic regression analysis are shown in Table 3, listing the statistical scores in descending order. Notably, the number of indicators used in the analysis has increased from 2 to 11. The combination method calculates the total by adding the largest statistical score to the second- and third-largest scores, and so on. The combined variable list is shown in Supplementary Table S1.

**Table 3:** Multivariate binary logistic regression analysis results.

Noninvasive parameter	S0 + S1 Vs. S2 + S3 + S4		S0 + S1 + S2 Vs. S3 + S4		S0 + S1 + S2 + S3 Vs. S4	
	Score	*p*-value	Score	*p*-value	Score	*p*-value
2D SWE	22.234	< 0.001**	50.218	< 0.001**	64.751	< 0.001**
Forns score	20.007	< 0.001**	37.378	< 0.001**	49.204	< 0.001**
FibroIndex	16.955	< 0.001**	32.851	< 0.001**	42.733	< 0.001**
APRI	6.239	0.012*	16.205	< 0.001**	31.208	< 0.001**
RPR	5.173	0.023*	12.769	< 0.001**	22.727	< 0.001**
CIV	4.752	0.029*	2.731	0.098	5.722	0.017*
King’s score	4.283	0.038*	12.511	< 0.001**	25.490	< 0.001**
PCIII	3.928	0.041*	2.979	0.084	9.748	0.002**
FIB-4	3.847	0.043*	11.412	0.001**	23.124	< 0.001**
LN	3.745	0.046*	4.153	0.034*	10.226	0.001**
Hepascore	3.625	0.047*	3.916	0.042*	5.882	0.015*
HA	1.208	0.272	1.709	0.191	12.610	< 0.001**

2D SWE, Two-dimensional shear wave elastography; APRI, Aspartate transaminase-to-platelet ratio index; FIB-4, Fibrosis index based on the four factors; RPR, Red cell distribution width-to-platelet ratio; CIV, Type IV collagen; HA, Hyaluronic acid.

**Correlation is significant at the 0.01 level (two-tailed).

*Correlation is significant at the 0.05 level (two-tailed).

**Figure 1:**
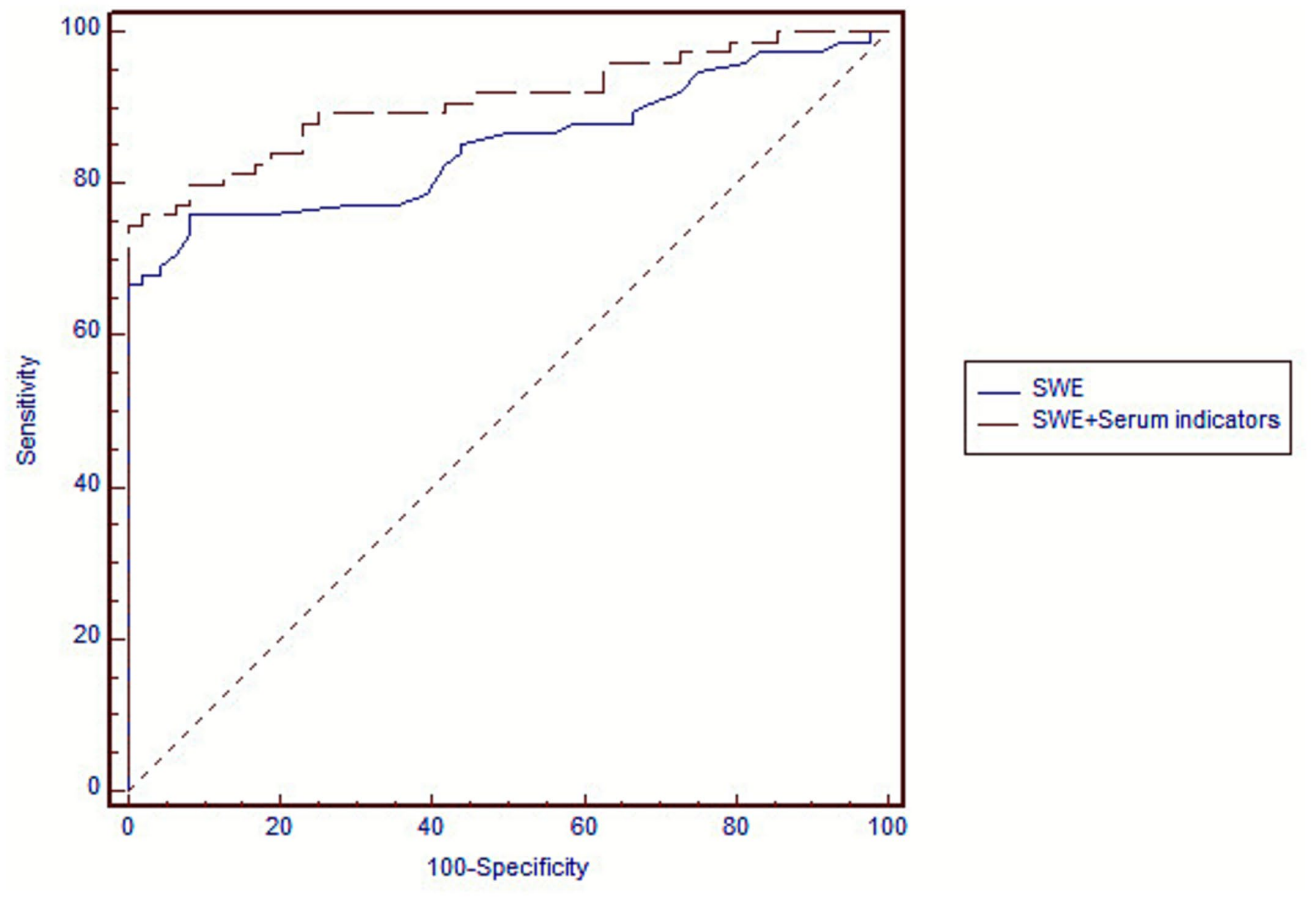
Receiver operating characteristic (ROC) curves showing performance in the diagnosis of substantial liver fibrosis (≥ S2) by two-dimensional shear wave elastography (2D SWE) and by 2D SWE combined with serum indicators. Diagonal segments are produced by ties.

2D SWE combined with a single serum marker did not improve the accuracy of diagnosing substantial liver fibrosis. The same was true for severe liver fibrosis and cirrhosis (Supplementary Table S2). Among the nine combined indicators for diagnosing substantial liver fibrosis, all had higher AUCs than 2D SWE alone. We selected 2D SWE combined with six serum indicators (five combined indicators) that had the fewest parameters to emphasize the trade-off between accuracy and simplicity. Combined indicator 5 increased the AUC for diagnosing substantial liver fibrosis from 0.802 to 0.889 (*p* = 0.040), with a sensitivity of 76.0% (67.7–85.8%) and a specificity of 97.9% (88.9–99.7%) (Figure 1). However, combining 2D SWE with multiple serum markers did not improve the accuracy of diagnosing severe liver fibrosis or cirrhosis (Table 4).

**Table 4:** 2D SWE combined with multiple serum indicators to diagnose liver fibrosis.

Combined indicators	Liver fibrosis stage ≥ S2				Liver fibrosis stage ≥ S3			
	AUC	*p*-value (combined markers vs. 2D SWE)	Sensitivity (%)	Specificity (%)	AUC	*p*-value (combined markers vs. 2D SWE)	Sensitivity (%)	Specificity (%)
2D SWE	0.805 (0.725–0.858)	/	75.0 (63.7–82.4)	90.5 (79.0–95.6)	0.945 (0.900–0.965)	/	92.3 (81.5–95.3)	90.5 (82.5–95.2)
Combined indicators 1	0.866 (0.792–0.920)	0.237	70.7 (59.0–80.6)	97.9 (88.9–99.7)	0.984 (0.943–0.998)	0.201	95.2 (83.8–99.4)	93.8 (86.2–98.0)
Combined indicators 2	0.866 (0.792–0.920)	0.237	70.7 (59.0–80.6)	97.9 (88.9–99.7)	0.984 (0.942–0.998)	0.203	95.2 (83.8–99.4)	93.8 (86.2–98.0)
Combined indicators 3	0.880 (0.809–0.931)	0.278	76.0 (64.7–85.1)	95.8 (85.7–99.4)	0.984 (0.940–0.997)	0.257	95.2 (83.8–99.3)	92.6 (84.6–97.2)
Combined indicators 4	0.874 (0.802–0.927)	0.143	73.3 (60.2–82.3)	97.9 (88.9–99.7)	0.984 (0.943–0.998)	0.317	95.2 (83.8–99.3)	95.1 (87.8–98.6)
Combined indicators 5	0.889 (0.820–0.939)	0.040*	76.0 (64.4–85.8)	97.9 (88.9–99.7)	0.982 (0.940–0.997)	0.432	95.2 (83.8–99.3)	95.1 (87.8–98.6)
Combined indicators 6	0.891 (0.822–0.940)	0.038*	76.0 (64.7–85.1)	95.8 (85.7–99.4)	0.982 (0.940–0.997)	0.521	95.2 (83.8–99.3)	95.1 (87.8–98.6)
Combined indicators 7	0.900 (0.833–0.947)	0.020*	70.7 (59.0–80.6)	100.0 (92.5–100.0)	0.982 (0.940–0.997)	0.521	95.2 (83.8–99.3)	95.1 (87.8–98.6)
Combined indicators 8	0.900 (0.831–0.946)	0.024*	70.7 (59.0–80.6)	100.0 (92.5–100.0)	0.986 (0.945–0.999)	0.321	97.6 (87.4–99.9)	96.3 (89.6–99.2)
Combined indicators 9	0.907 (0.841–0.952)	0.012*	76.0 (64.7–85.1)	95.8 (85.7–99.4)	0.986 (0.945–0.999)	0.321	97.6 (87.4–99.9)	96.3 (89.6–99.2)

Combined indicators	Liver fibrosis stage S4			
	AUC	*p*-value (combined markers vs. 2D SWE)	Sensitivity (%)	Specificity (%)
2D SWE	0.970 (0.922–0.991)	/	90.3 (69.9–95.5)	85.0 (78.3.-90.0)
Combined indicators 1	0.995 (0.961–0.998)	0.097	95.7 (78.0–99.3)	97.0 (91.5–99.4)
Combined indicators 2	0.995 (0.961–0.998)	0.082	95.7 (78.0–99.3)	98.0 (92.9–99.7)
Combined indicators 3	0.996 (0.963–0.997)	0.078	95.7 (78.0–99.3)	99.0 (94.5–99.8)
Combined indicators 4	0.999 (0.968–1.00)	0.065	100.0 (85.0–100.0)	97.0 (91.5–99.3)
Combined indicators 5	1.000 (0.970–1.00)	0.062	100.0 (85.0–100.0)	100.0 (96.3–100.0)
Combined indicators 6	1.000 (0.970–1.00)	0.062	100.0 (85.0–100.0)	100.0 (96.3–100.0)
Combined indicators 7	1.000 (0.970–1.00)	0.062	100.0 (85.0–100.0)	100.0 (96.3–100.0)
Combined indicators 8	1.000 (0.970–1.00)	0.062	100.0 (85.0–100.0)	100.0 (96.3–100.0)
Combined indicators 9	1.000 (0.970–1.00)	0.062	100.0 (85.0–100.0)	100.0 (96.3–100.0)

AUC, area under the receiver operating characteristic curve; 2D SWE, two-dimensional shear wave elastography; APRI, aspartate transaminase-to-platelet ratio index; FIB-4, fibrosis index based on the four factors; RPR, red cell distribution width-to-platelet ratio; PCIII, procollagen type III; CIV, collagen type IV; LN, laminin; HA, hyaluronic acid.

Combined indicators 1: 2D SWE + Forns score + FibroIndex; Combined indicators 2: 2D SWE + Forns score + FibroIndex + ARPI; Combined indicators 3: 2D SWE + Forns score + FibroIndex + ARPI + RPR; Combined indicators 4: 2D SWE + Forns score + FibroIndex + ARPI + RPR + CIV; Combined indicators 5: 2D SWE + Forns score + FibroIndex + ARPI + RPR + CIV + King’s score; Combined indicators 6: 2D SWE + Forns score + FibroIndex + ARPI + RPR + CIV + King’s score + PCIII; Combined indicators 7: 2D SWE + Forns score + FibroIndex + ARPI + RPR + CIV + King’s score + PCIII + FIB-4; Combined indicators 8: 2D SWE + Forns score + FibroIndex + ARPI + RPR+ CIV + King’s score + PCIII + FIB-4 + LN; Combined indicators 9: 2D SWE + Forns score + FibroIndex + ARPI + RPR + CIV + King’s score + PCIII + FIB-4 + LN + Hepascore.

*Correlation is significant at the 0.05 level (two-tailed).

### Evaluation of liver fibrosis stage using multiple serum indicators

**Figure 2:**
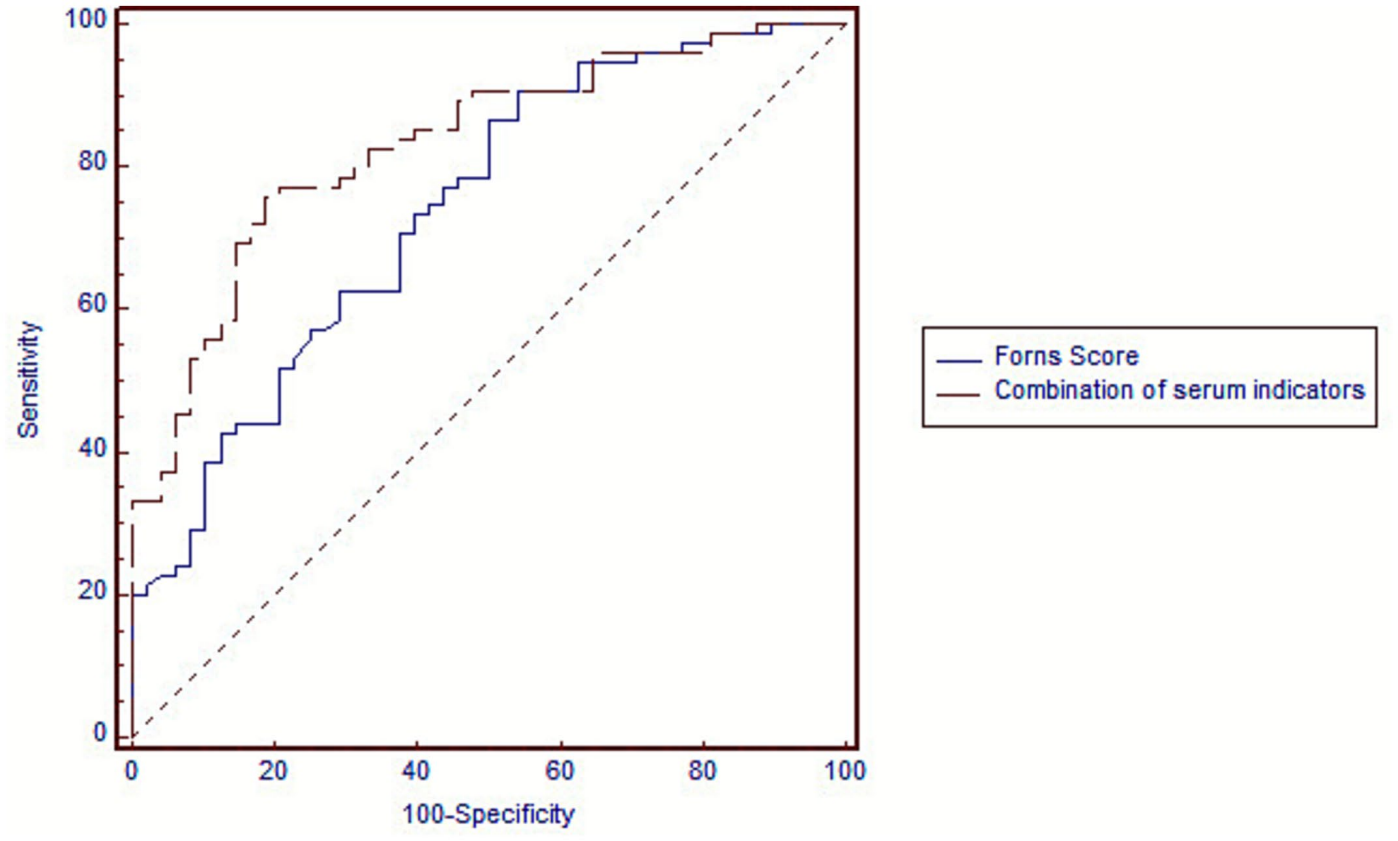
Receiver operating characteristic (ROC) curves showing performance in the diagnosis of substantial liver fibrosis (≥ S2) using the Forns score and its combination of serum indicators. Diagonal segments are produced by ties.

Among the 11 serum indicators, the Forns score showed the strongest correlation with liver fibrosis (r = 0.566, *p* < 0.001). For substantial liver fibrosis, the AUC was 0.735, with a sensitivity of 80.7% and a specificity of 53.0%. For severe liver fibrosis, the AUC was 0.829, with a sensitivity of 92.9% and a specificity of 60.5%. For cirrhosis, the AUC was 0.909, with a sensitivity of 69.6% and a specificity of 99.0%. When the Forns score was combined with the FibroIndex, APRI, RPR, CIV, King’s score, PCIII, FIB-4, LN, and Hepascore, the AUC for diagnosing substantial liver fibrosis increased from 0.739 to 0.825 (*p* = 0.016), with a sensitivity of 85.3% and a specificity of 62.5%; for severe liver fibrosis, the AUC increased from 0.815 to 0.881 (*p* = 0.024), with a sensitivity of 92.9% and a specificity of 75.3%; for cirrhosis, the AUC rose from 0.904 to 0.954 (*p* = 0.013), with a sensitivity of 91.3% and a specificity of 86.0% (Figure 2; Table 5).

**Table 5:** The combination of 10 serum indicators in the diagnosis of liver fibrosis.

Combined indicators	Liver fibrosis stage ≥ S2				Liver fibrosis stage ≥ S3			
	AUC	*p*-value (combined markers vs. Forns score)	Sensitivity (%)	Specificity (%)	AUC	*p*-value (combined markers vs. Forns score)	Sensitivity (%)	Specificity (%)
Forns score	0.735 (0.650–0.810)	/	80.7 (72.8–91.4)	53.0 (38.2–65.8)	0.815 (0.730–0.881)	/	91.5 (78.5–95.4)	65.5 (53.0–75.8)
Combined serum indicators 1	0.740 (0.653–0.815)	0.922	74.7 (63.7–84.3)	64.6 (49.5–77.8)	0.833 (0.755–0.894)	0.629	71.4 (55.4–84.3)	84.0 (74.1–91.2)
Combined serum indicators 2	0.754 (0.668–0.827)	0.245	78.7 (66.2–83.3)	66.7 (51.5–79.8)	0.838 (0.761–0.898)	0.373	88.1 (74.4–96.0)	65.4 (54.0–75.7)
Combined serum indicators 3	0.755 (0.669–0.828)	0.239	77.3 (66.2–86.2)	66.7 (51.6–79.6)	0.842 (0.766–0.902)	0.189	88.1 (74.4–96.0)	67.9 (56.6–77.8)
Combined serum indicators 4	0.766 (0.681–0.837)	0.207	75.3 (64.2–84.2)	66.5 (52.6–70.6)	0.845 (0.769–0.904)	0.120	92.7 (80.5–98.5)	63.0 (51.5–73.4)
Combined serum indicators 5	0.783 (0.700–0.852)	0.127	81.3 (70.7–89.4)	64.6 (49.5–77.8)	0.848 (0.772–0.904)	0.123	92.9 (80.5–98.5)	64.2 (52.8–74.6)
Combined serum indicators 6	0.785 (0.702–0.854)	0.108	82.7 (72.2–90.4)	62.5 (47.4–76.0)	0.856 (0.781–0.913)	0.052	71.4 (55.4–84.3)	87.7 (78.5–93.9)
Combined serum indicators 7	0.787 (0.704–0.856)	0.092	58.7 (46.7–69.9)	87.5 (74.7–95.2)	0.856 (0.781–0.913)	0.056	71.4 (55.4–84.3)	86.4 (77.0–93.0)
Combined serum indicators 8	0.792 (0.709–0.860)	0.074	82.7 (72.2–90.4)	62.5 (47.4–76.0)	0.880 (0.807–0.930)	0.021*	92.9 (80.5–98.4)	74.4 (63.1–83.2)
Combined serum indicators 9	0.825 (0.746–0.888)	0.016*	85.3 (75.3–92.4)	62.5 (47.4–76.0)	0.881 (0.810–0.932)	0.024*	92.9 (80.5–98.4)	75.3 (64.5–84.2)

Combined indicators	Liver fibrosis stage S4			
	AUC	*p*-value (combined markers vs. Forns score)	Sensitivity (%)	Specificity (%)
Forns score	0.904 (0.823–0.923)	/	69.6 (48.1–88.7)	94.0 (90.5–97.8)
Combined serum indicators 1	0.933 (0.874–0.974)	0.053	91.3 (72.0–98.9)	79.0 (69.7–86.5)
Combined serum indicators 2	0.946 (0.890–0.978)	0.020*	78.3 (56.3–92.5)	99.0 (94.5–100.0)
Combined serum indicators 3	0.946 (0.890–0.979)	0.042*	73.9 (51.6–89.8)	100.0 (96.4–100.0)
Combined serum indicators 4	0.945 (0.888–0.978)	0.048*	73.9 (51.6–89.8)	100.0 (96.4–100.0)
Combined serum indicators 5	0.947 (0.890–0.979)	0.038*	73.9 (51.6–89.8)	100.0 (96.4–100.0)
Combined serum indicators 6	0.952 (0.898–0.982)	0.023*	100.0 (85.2–100.0)	75.0 (65.3–83.1)
Combined serum indicators 7	0.948 (0.892–0.980)	0.029*	78.3 (56.3–95.2)	95.0 (88.7–98.4)
Combined serum indicators 8	0.950 (0.896–0.981)	0.015*	91.3 (72.0–98.9)	84.0 (75.3–90.6)
Combined serum indicators 9	0.954 (0.900–0.984)	0.013*	91.3 (72.0–98.9)	86.0 (77.6–92.1)

AUC, area under the receiver operating characteristic curve; APRI, aspartate transaminase-to-platelet ratio index; FIB-4, fibrosis index based on the four factors; RPR, red cell distribution width-to-platelet ratio; PCIII, procollagen type III; CIV, collagen type IV; LN, laminin; HA, hyaluronic acid.

Combined serum indicators 1: Forns score + FibroIndex; Combined serum indicators 2: Forns score + FibroIndex + ARPI; Combined serum indicators 3: Forns score + FibroIndex + ARPI + RPR; Combined serum indicators 4: Forns score + FibroIndex + ARPI + RPR + CIV; Combined serum indicators 5: Forns score + FibroIndex + ARPI + RPR + CIV + King’s score; Combined serum indicators 6: Forns score + FibroIndex + ARPI + RPR + CIV + King’s score + PCIII; Combined serum indicators 7: Forns score + FibroIndex + ARPI + RPR + CIV + King’s score + PCIII + FIB-4; Combined serum indicators 8: Forns score+ FibroIndex + ARPI+ RPR + CIV + King’s score + PCIII + FIB-4 + LN; Combined serum indicators 9: Forns score + FibroIndex + ARPI + RPR + CIV + King’s score + PCIII + FIB-4 + LN + Hepascore.

*Correlation is significant at the 0.05 level (two-tailed).

### Diagnostic performance of effective combined indicators for assessing liver fibrosis stages in training and validation cohorts

The effective combined indicator 5 (2D SWE + Forns score + FibroIndex + ARPI + RPR + CIV + King’s score) diagnosed substantial liver fibrosis in both the training and validation cohorts, with scores of 0.887 and 0.881, respectively. The effective serum combined indicator 9 (Forns score + FibroIndex + ARPI + RPR + CIV + King’s score + PCIII + FIB-4 + LN + Hepascore) diagnosed substantial liver fibrosis, severe liver fibrosis, and cirrhosis in both the training and validation cohorts, with scores of 0.817 and 0.813, 0.879 and 0.884, and 0.949 and 0.951, respectively (Table 6).

**Table 6:** Diagnostic performance of effective combined indicators for assessing liver fibrosis stages in training and validation cohorts.

Liver fibrosis stage	Training cohort (*n* = 61)			Validation cohort (*n* = 41)		
	AUC	Sensitivity (%)	Specificity (%)	AUC	Sensitivity (%)	Specificity (%)
Combined indicators 5						
Liver fibrosis stage ≥S2	0.887 (0.812–0.935)	73.0 (62.5–84.7)	95.8 (88.4–99.5)	0.881 (0.816–0.930)	74.0 (63.8–85.4)	96.5 (89.4–99.7)
Combined serum indicators 9						
Liver fibrosis stage ≥S2	0.817 (0.738–0.880)	83.3 (73.4–90.4)	65.5 (50.4–79.0)	0.813 (0.734–0.876)	81.0 (71.3–92.4)	50.5 (40.4–69.0)
Liver fibrosis stage ≥S3	0.879 (0.807–0.929)	91.8 (79.5–97.3)	75.5 (64.7–86.4)	0.884 (0.816–0.935)	88.8 (74.5–97.5)	76.5 (65.6–89.4)
Liver fibrosis stage S4	0.949 (0.897–0.983)	90.2 (71.0–97.8)	87.0 (78.5–93.1)	0.951 (0.899–0.988)	92.2 (76.0–97.9)	83.2 (72.3–95.1)

AUC, area under the receiver operating characteristic curve.

Combined indicators 5: 2D SWE + Forns score + FibroIndex + ARPI + RPR + CIV + King’s score.

Combined serum indicators 9: Forns score + FibroIndex + ARPI + RPR + CIV+ King’s score + PCIII + FIB-4 + LN + Hepascore.

*Correlation is significant at the 0.05 level (two-tailed).

## Discussion

This study enhanced the diagnostic performance for liver fibrosis by combining serum markers with 2D SWE, increasing the AUC from 0.805 to 0.889 and demonstrating a marked improvement in accuracy. This advancement may significantly influence clinical decision-making, as the higher AUC reflects improved detection of significant liver fibrosis, enabling earlier intervention, preventing disease progression, and potentially guiding updates to clinical guidelines and screening strategies.

In contrast to previous studies by Zhuang et al. (29) and Liu et al. (30), which separately evaluated 2D SWE and serum models and reported divergent diagnostic efficacy, possibly due to differences in study populations, our study innovatively integrates 2D SWE with multiple serum models. This integrated approach yields superior diagnostic performance for significant liver fibrosis compared with Liu’s results (AUC 0.889 vs. 0.851).

The two methodologies provide complementary information through distinct mechanisms: 2D SWE quantifies liver stiffness, with elevated values indicating more advanced fibrosis or cirrhosis, while serum markers reflect liver functional status and injury-related inflammation, which correlate with fibrosis staging.

Although 2D SWE is susceptible to sampling errors due to limited measurement areas and heterogeneous fibrosis distribution, serum biomarkers offer a more comprehensive assessment of hepatic condition. The combined approach compensates for SWE’s limitations in detecting mild fibrosis by enabling the identification of significant fibrosis even in patients with normal serum markers, thereby expanding coverage across the spectrum of liver fibrosis. In our study, we observed no significant correlation between HA and the stages of liver fibrosis. This was not the result we expected, as previous literature has reported a correlation between HA and liver fibrosis (33, 34). This indicates that the correlation between HA and liver fibrosis in patients with hepatitis B is controversial. Our study showed that adding a single serum marker to SWE did not significantly enhance diagnostic accuracy. This may be due to overlap in the information provided by the two techniques. When serum markers closely relate to the same pathological processes assessed by 2D SWE, much of the information overlaps. The small differences between them are insufficient to provide additional diagnostic value. Therefore, we decided to combine multiple indicators to highlight these minor differences and create significant distinctions.

Previous studies have shown that 2D SWE combined with the FIB-4 score can improve the accuracy of diagnosing substantial and severe liver fibrosis in patients with hepatitis C (35). However, in our study, 2D SWE combined with FIB-4 did not improve the accuracy of liver fibrosis staging in patients with CHB. This may be due to differences between chronic hepatitis B and hepatitis C-related fibrosis. Research indicates that HBV infection causes direct hepatocyte damage and triggers immune-mediated inflammatory responses that promote liver fibrosis progression. In contrast, fibrosis progression in HCV infection is more closely associated with chronic inflammation and ongoing hepatocyte damage (36).

AST and ALT levels are commonly used to assess the severity of hepatocyte injury. Antiviral treatment can be started when these levels exceed the normal range by more than twofold. However, HBV patients with normal AST or ALT levels may still experience substantial liver fibrosis. In fact, substantial liver fibrosis is one of the indicators that justify antiviral treatment (37, 38). Although various serum liver fibrosis indicators and imaging techniques are emerging as high-value tools for evaluating severe liver fibrosis and cirrhosis, their effectiveness in assessing substantial liver fibrosis is comparatively lower. To date, no single indicator is sufficiently sensitive and accurate to reflect substantial liver fibrosis and replace a liver biopsy. In this study, we found that the combination of noninvasive markers significantly increased diagnostic accuracy for identifying substantial liver fibrosis and provided a crucial foundation for the clinical decision to initiate antiviral therapy.

The high cost of 2D SWE-equipped ultrasound equipment poses a barrier to its accessibility in many medical institutions, thereby limiting its widespread clinical use in conjunction with serum markers. In contrast, the necessary equipment for serum marker testing is typically available in primary hospitals, and the associated testing costs are relatively low. Importantly, our findings demonstrate that combining 10 serum markers can enhance diagnostic accuracy for significant, severe liver fibrosis and cirrhosis. The model based solely on these serum markers shows an AUC for diagnosing significant liver fibrosis and cirrhosis that is comparable to that of 2D SWE, underscoring its potential as a cost-effective alternative in low-resource settings.

This study also compared the value of 2D SWE and VCTE in diagnosing liver fibrosis in a cohort of CHB patients and found that there was no significant difference in AUC between 2D SWE and VCTE for diagnosing substantial liver fibrosis, severe liver fibrosis, and cirrhosis. This aligns with recent findings that both methods are equally effective in diagnosing liver fibrosis in patients with chronic liver diseases, including hepatitis B and C (39, 40). However, another previous study showed that 2D SWE was superior to VCTE in diagnosing substantial liver fibrosis in patients with hepatitis C (41). This suggests that 2D SWE and VCTE may have different diagnostic performances in assessing liver fibrosis in patients with varying causes. Additionally, 2D SWE, which utilizes real-time two-dimensional ultrasound guidance, enables accurate localization of sampling sites. This method can be effectively employed in patients with obesity, ascites, and narrow intercostal spaces, while VCTE presents certain limitations. In addition, the cutoff values for diagnosing liver fibrosis differ between these two methods.

In this study, an effective combination indicator model derived from the overall cohort improved the AUC for diagnosing liver fibrosis; however, given the limited sample size, there may be a risk of overfitting. To address this issue, we used internal validation in the analysis, and these effective combination indices showed the same diagnostic accuracy in the validation cohort. In diagnosing cirrhosis, some combination indicators (indicators 7–9) had an AUC of 1.00, and there was no statistically significant difference between the combination indicator and the single indicator (2D SWE) in diagnosing cirrhosis (1.00 vs. 0.97, *p* = 0.062); therefore, we consider these models to be ineffective combinations. Although high AUC values indicate model performance, they should be interpreted with caution, given the limited sample size.

The imbalanced distribution of fibrosis stages, especially the limited number of S0 patients (only 3), posed a challenge for this study. To address this, S0 and S1 patients were combined into a single group. However, this approach may introduce bias in performance estimates and limit the generalizability of the findings. Therefore, future studies with external or multicenter validation are necessary to strengthen the model’s robustness and broader applicability.

There are also several limitations in this study. First, the relatively small sample size of this study may affect the generalizability of our findings, particularly for S3 and S4 patients. Second, the strong correlation and close linear relationship between histological fibrosis staging and elastography may overestimate the model’s predictive performance. Third, this study is limited to chronic hepatitis B, and its significance for liver fibrosis caused by other diseases warrants further discussion. Fourth, some of the included blood-based markers are not widely available, and the complex calculations of combined indicators limit the use of the current study’s results in routine clinical practice. In the future, we will develop an AI-driven decision-support system to assist clinicians in interpreting complex models.

In conclusion, 2D SWE and VCTE have equivalent diagnostic accuracy for liver fibrosis in patients with CHB. The combination of multiple noninvasive indicators can improve the accuracy of liver fibrosis diagnosis. This approach offers a potential noninvasive alternative to liver biopsy for assessing liver fibrosis, particularly in advanced stages requiring clinical intervention.

## Data Availability

The raw data supporting the conclusions of this article will be made available by the authors, without undue reservation.
