# Supplementary material for: Non-invasive assessment of liver fibrosis staging in chronic hepatitis B patients: combining two-dimensional shear wave elastography with serum indicators
Source: Front Med (Lausanne). 2026 Jan 12;12:1709007. doi: 10.3389/fmed.2025.1709007 (PMC12833436; doi:10.3389/fmed.2025.1709007)
Supplement: Supplementary file 1 [file Data_Sheet_1.docx]

Supplementary Table S1

 Models involved in various combined indicators

| Combined indicators | Involved models |
| --- | --- |
| Combined indicators 1 | 2D SWE + Forns score + FibroIndex |
| Combined indicators 2 | 2D SWE + Forns score + FibroIndex + ARPI |
| Combined indicators 3 | 2D SWE + Forns score + FibroIndex + ARPI + PRP |
| Combined indicators 4 | 2D SWE + Forns score + FibroIndex + ARPI + PRP + CIV |
| Combined indicators 5 | 2D SWE + Forns score + FibroIndex + ARPI + PRP + CIV + King’s score |
| Combined indicators 6 | 2D SWE + Forns score + FibroIndex + ARPI + PRP + CIV + King’s score + PCⅢ |
| Combined indicators 7 | 2D SWE + Forns score + FibroIndex + ARPI + PRP + CIV + King’s score + PCⅢ + FIB-4 |
| Combined indicators 8 | 2D SWE + Forns score + FibroIndex + ARPI + PRP+ CIV + King’s score + PCⅢ + FIB-4 + LN |
| Combined indicators 9 | 2D SWE + Forns score + FibroIndex + ARPI + PRP + CIV + King’s score + PCⅢ + FIB-4 + LN + Hepascore. |
| Serum Combined indicators | |
| Serum Combined indicators 1 | Forns score + FibroIndex |
| Serum Combined indicators 2 | Forns score + FibroIndex + ARPI |
| Serum Combined indicators 3 | Forns score + FibroIndex + ARPI + PRP |
| Serum Combined indicators 4 | Forns score + FibroIndex + ARPI + PRP + CIV |
| Serum Combined indicators 5 | Forns score + FibroIndex + ARPI + PRP + CIV + King’s score |
| Serum Combined indicators 6 | Forns score + FibroIndex + ARPI + PRP + CIV + King’s score + PCⅢ |
| Serum Combined indicators 7 | Forns score + FibroIndex + ARPI + PRP + CIV + King’s score + PCⅢ + FIB-4 |
| Serum Combined indicators 8 | Forns score+ FibroIndex + ARPI+ PRP + CIV + King’s score + PCⅢ + FIB-4 + LN |
| Serum Combined indicators 9 | Forns score + FibroIndex + ARPI + PRP + CIV + King’s score + PCⅢ + FIB-4 + LN + Hepascore |

Supplementary Table S2

2D SWE combined with a single serum index to diagnose liver fibrosis.

| Combined indicators | Liver fibrosis stage ≥ S2 | | | | Liver fibrosis stage ≥ S3 | | | |
| --- | --- | --- | --- | --- | --- | --- | --- | --- |
|  | AUC | *P*-value  （combined markers vs. 2D SWE） | Sensitivity (%) | Specificity (%) | AUC | *P-value*  （combined markers vs. 2D SWE） | Sensitivity (%) | Specificity (%) |
| 2D SWE | 0.805 (0.725-0.858) | / | 75.0 (63.7-82.4) | 90.5(79.0-95.6) | 0.945 (0.900-0.965) | / | 92.3 (81.5-95.3.) | 90.5 (82.5-95.2) |
| 2D SWE+Forns score | 0.816 (0.743-0.871) | 0.240 | 70.7 (59.0-80.6) | 97.9 (88.9-99.7) | 0.944 (0.913-0.968) | 0.200 | 92.9 (79.5-98.4) | 93.7 (85.8-96.6) |
| 2D SWE + APRI | 0.806 (0.731-0.863) | 0.558 | 72.0 (60.4-81.8) | 97.9 (88.9-99.7) | 0.949 (0.906-0.966) | 0.355 | 95.2 (81.5-95.3) | 91.8 (84.2-95.9) |
| 2D SWE + King’s score | 0.802 (0.726-0.859) | 0.797 | 73.3 (61.9-82.9) | 95.8 (85.7-99.4) | 0.949 (0.906-0.966) | 0.395 | 95.2 (81.5-95.3) | 93.7 (85.8-96.6) |
| 2D SWE + FibroIndex | 0.813 (0.739-0.868) | 0.279 | 70.7(59.0-80.6) | 100.0 (92.5-100.0) | 0.953 (0.911-0.967) | 0.243 | 95.2 (81.5-95.3) | 93.1 (82.8-96.6) |
| 2D SWE + FIB-4 | 0.800 (0.725-0.858) | 0.931 | 74.7 (63.3-84.0) | 93.8 (82.8-98.6) | 0.949 (0.906-0.966) | 0.423 | 95.2 (81.5-95.3) | 93.1 (85.8-96.6) |
| 2D SWE +RPR | 0.814 (0.740-0.869) | 0.255 | 70.7(59.0-80.6) | 100.0 (92.5-100.0) | 0.941 (0.908-0.967) | 0.312 | 95.2 (81.5-95.3) | 93.1 (85.8-96.6) |
| 2D SWE + CIV | 0.812 (0.738-0.867) | 0.326 | 70.7 (59.0-80.6) | 97.9 (88.9-99.7) | 0.945 (0.899-0.964) | 0.716 | 95.2 (81.5-95.3) | 90.5 (82.5-95.2) |
| 2D SWE + LN | 0.813 (0.740-0.869) | 0.261 | 74.7 (63.3-84.0) | 93.8 (82.8-98.6) | 0.945 (0.899-0.964) | 0.717 | 95.2 (81.5-95.3) | 90.5 (82.5-95.2) |
| 2D SWE + HA | 0.802 (0.727-0.860) | 0.397 | 73.3 (61.9-82.9) | 93.8 (82.8-98.6) | 0.945 (0.899-0.964) | 0.717 | 95.2 (81.5-95.3) | 90.5 (82.5-95.2) |
| 2D SWE + PCIII | 0.811 (0.737-0.867) | 0.210 | 69.3 (57.6-79.5) | 95.8 (85.7-99.4) | 0.945 (0.899-0.964) | 0.529 | 95.2 (81.5-95.3) | 90.5 (82.5-95.2) |
| 2D SWE + Hepascore | 0.810 (0.736-0.866) | 0.249 | 69.3 (57.6-79.5) | 100.0 (92.5-100.0) | 0.946 (0.900-0.965) | 0.835 | 92.7 79.5-98.4) | 90.5 (82.5-95.2) |

Supplementary Table S2 (Continued)

| Combined indicators | Liver fibrosis stage S4 | | | |
| --- | --- | --- | --- | --- |
|  | AUC | *P-value*  （combined markers vs. 2D SWE） | Sensitivity (%) | Specificity (%) |
| 2D SWE | 0.970 (0.922-0.991) | / | 90.3 (69.9-95.5) | 85.0 (78.3.-90.0) |
| 2D SWE+Forns score | 0.994 (0.959-0.998) | 0.110 | 100.0 (85.0-100.0) | 93.0 (86.1-97.1) |
| 2D SWE + APRI | 0.993 (0.957-0.998) | 0.131 | 95.7 (78.0-99.3) | 96.0 (90.1-98.9) |
| 2D SWE + King’s score | 0.993 (0.957-0.998) | 0.133 | 95.7 (78.0-99.3) | 97.0 (91.5-99.3) |
| 2D SWE + FibroIndex | 0.993 (0.957-0.998) | 0.008 | 95.7 (78.0-99.3) | 96.0 (90.1-98.9) |
| 2D SWE + FIB-4 | 0.992 (0.955-0.999) | 0.151 | 95.7 (78.0-99.3) | 96.0 (90.1-98.9) |
| 2D SWE +RPR | 0.979 (0.935-0.996) | 0.354 | 95.7 (78.0-99.3) | 88.0 (80.0-93.6) |
| 2D SWE + CIV | 0.980 (0.937-0.997) | 0.115 | 95.7 (78.0-99.3) | 90.0 (82.4-95.1) |
| 2D SWE + LN | 0.981 (0.939-0.997) | 0.241 | 95.7 (78.0-99.3) | 94.0 (87.4-97.8) |
| 2D SWE + HA | 0.980 (0.936-0.996) | 0.287 | 95.7 (78.0-99.3) | 90.0 (82.4-95.1) |
| 2D SWE + PCIII | 0.980 (0.936-0.996) | 0.410 | 95.7 (78.0-99.3) | 91.0 (83.6-95.8) |
| 2D SWE + Hepascore | 0.973 (0.927-0.994) | 0.735 | 95.7 (78.0-99.3) | 88.0 (80.0-93.6) |

AUC = area under receiver operating characteristic curve, 2D SWE = two-dimensional shear wave elastography, APRI = aspartate transaminase–to-platelet ratio index, FIB-4 = fibrosis index based on the four factors, RPR = red cell distribution width-to-platelet ratio, PCⅢ = procollagen type III, CⅣ = collagen type IV, LN= laminin, HA = hyaluronic acid
